# Supplementary material for: Optimization of 2-Aminoquinazolin-4-(3H)-one Derivatives as Potent Inhibitors of SARS-CoV-2: Improved Synthesis and Pharmacokinetic Properties
Source: Pharmaceuticals (Basel). 2022 Jul 4;15(7):831. doi: 10.3390/ph15070831 (PMC9318802; doi:10.3390/ph15070831)
Supplement: Supplementary file 1 [file pharmaceuticals-15-00831-s001.zip › pharmaceuticals-1745536-supplementary.pdf]

Supporting Information

Optimization of 2-aminoquinazolin-4(3*H*)-one derivatives as potent inhibitors of SARS-CoV-2: efficient synthesis and improved pharmacokinetic properties

Young Sup Shin[1,2,†], Jun Young Lee[1,2,†], Sangeun Jeon[3], Jung-Eun Cho[2], Subeen Myung[2,4], Min Seong Jang[5], Seungtaek Kim[3], Jong Hwan Song[2], Hyoung Rae Kim[2], Hyeung-geun Park[1,*], Lak Shin Jeong[1,*], Chul Min Park[2,4,*]

[1]*Research Institute of Pharmaceutical Sciences, College of Pharmacy, Seoul National University, Seoul 08826, Korea*

[2]*Center for Convergent Research of Emerging Virus Infection (CEVI), Korea Research Institute of Chemical Technology, 141 Gajeong-ro, Yuseong-gu, Daejeon 34114, Korea*

[3]*Zoonotic Virus Laboratory, Institut Pasteur Korea, Seongnam-si, Gyeonggi-do 13488, Korea*

[4]*Medicinal Chemistry and Pharmacology, Korea University of Science and Technology, Daejeon 34114, Korea*

[5]*Department of Non-Clinical Studies, Korea Institute of Toxicology, Yuseong-gu, Daejeon 34114, Korea*

[*]Correspondence: parkcm@krict.re.kr (C.M. Park), lakjeong@snu.ac.kr (L.S. jeong), hgpk@snu.ac.kr (H.-g. Park)

[†] These authors contributed equally to this work

†

Table of contents:

^{1}H&^{13}C NMR Spectra for **1a**……………………………………………………………S3

^{1}H&^{13}C NMR Spectra for **1b**……………………………………………………………S4

^{1}H&^{13}C NMR Spectra for **1c**……………………………………………………………S5

^{1}H&^{13}C NMR Spectra for **4a**……………………………………………………………S6

^{1}H&^{13}C NMR Spectra for **4b**……………………………………………………………S7

^{1}H&^{13}C NMR Spectra for **5c**……………………………………………………………S8

^{1}H&^{13}C NMR Spectra for **2a**……………………………………………………………S9

^{1}H&^{13}C NMR Spectra for **2b**…………………………………………………………..S10

^{1}H&^{13}C NMR Spectra for **2c**…………………………………………………………..S11

^{1}H&^{13}C NMR Spectra for **6a**…………………………………………………………..S12

^{1}H&^{13}C NMR Spectra for **6b**…………………………………………………………..S13

^{1}H&^{13}C NMR Spectra for **6c**…………………………………………………………..S14

^{1}H&^{13}C NMR Spectra for **6d**…………………………………………………………..S15

^{1}H&^{13}C NMR Spectra for **7**……………………………………………………………S16

^{1}H&^{13}C NMR Spectra for **8**……………………………………………………………S17

Figure 1S. Concentration-response inhibition curves of **2a** and **2b** against SARS-CoV-2 pseudovirus…………………………………………………………………………S18

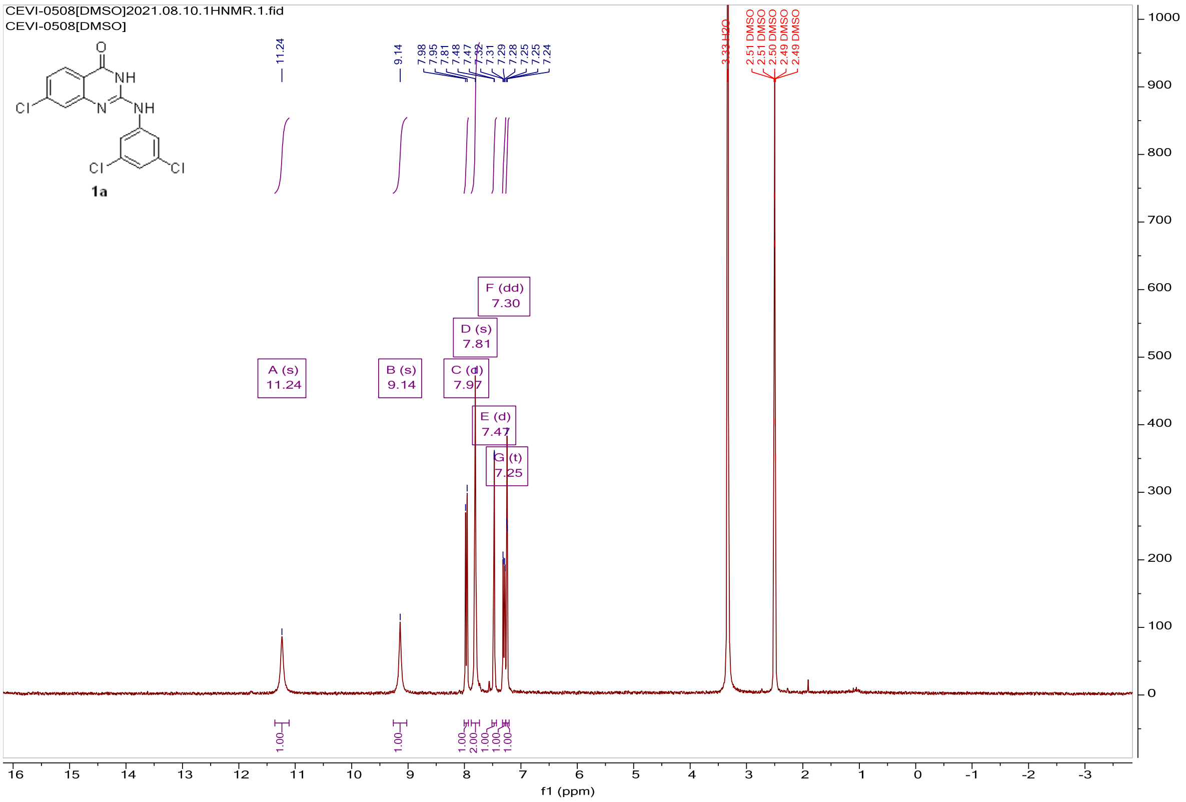

^{1}H NMR spectrum (400 MHz, DMSO-d_6) of **1a**

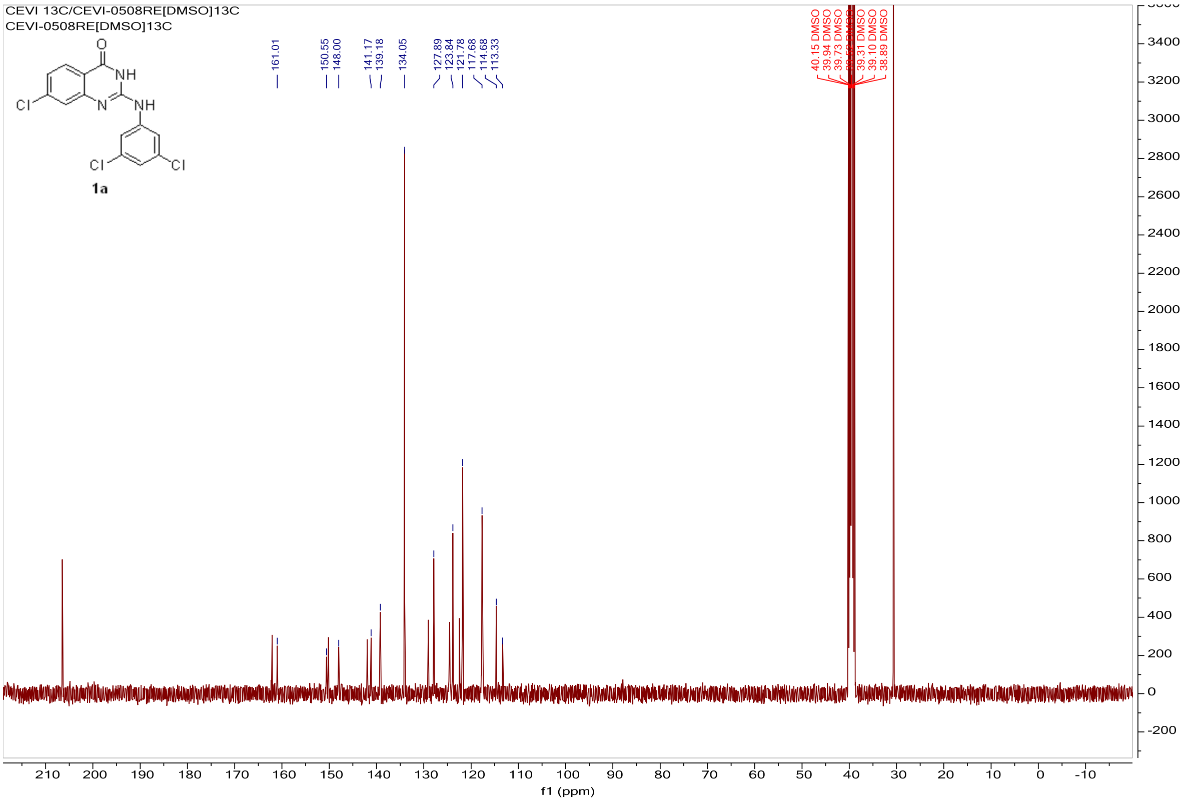

^{13}C NMR spectrum (100 MHz, DMSO-d_6) of **1a**

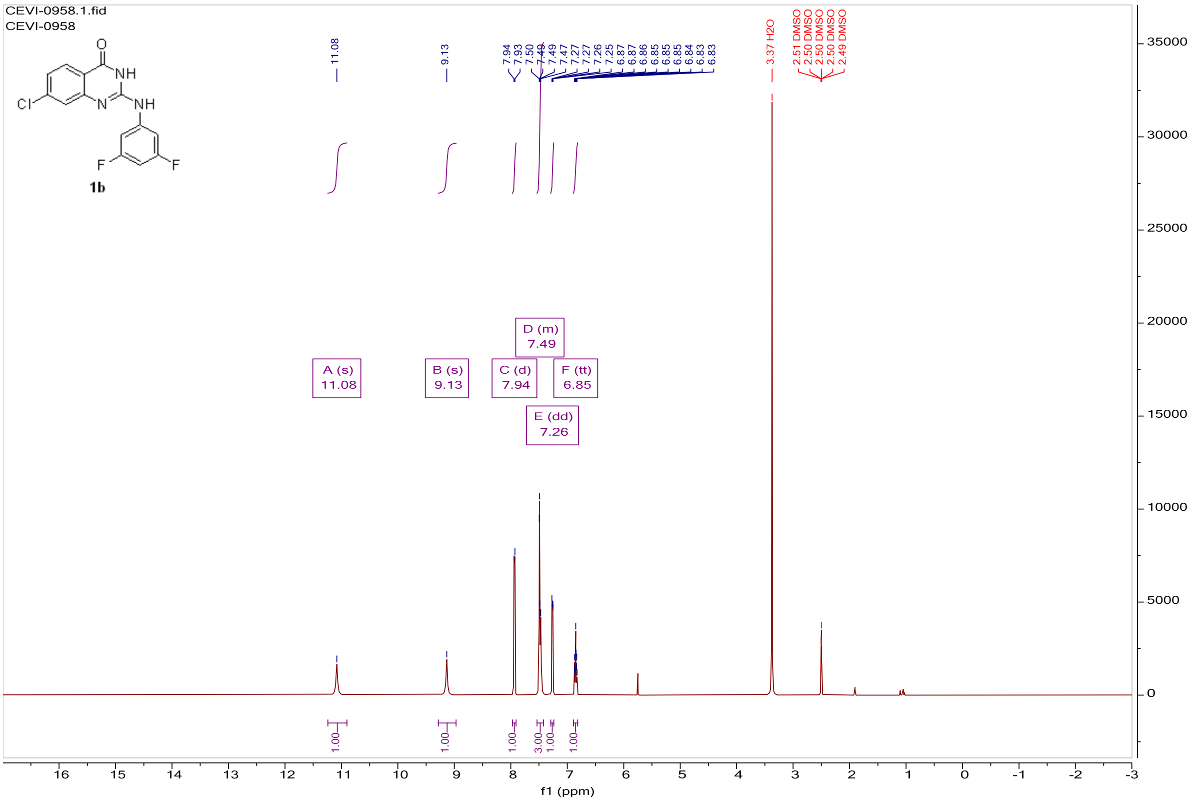

^{1}H NMR spectrum (500 MHz, DMSO-d_6) of **1b**

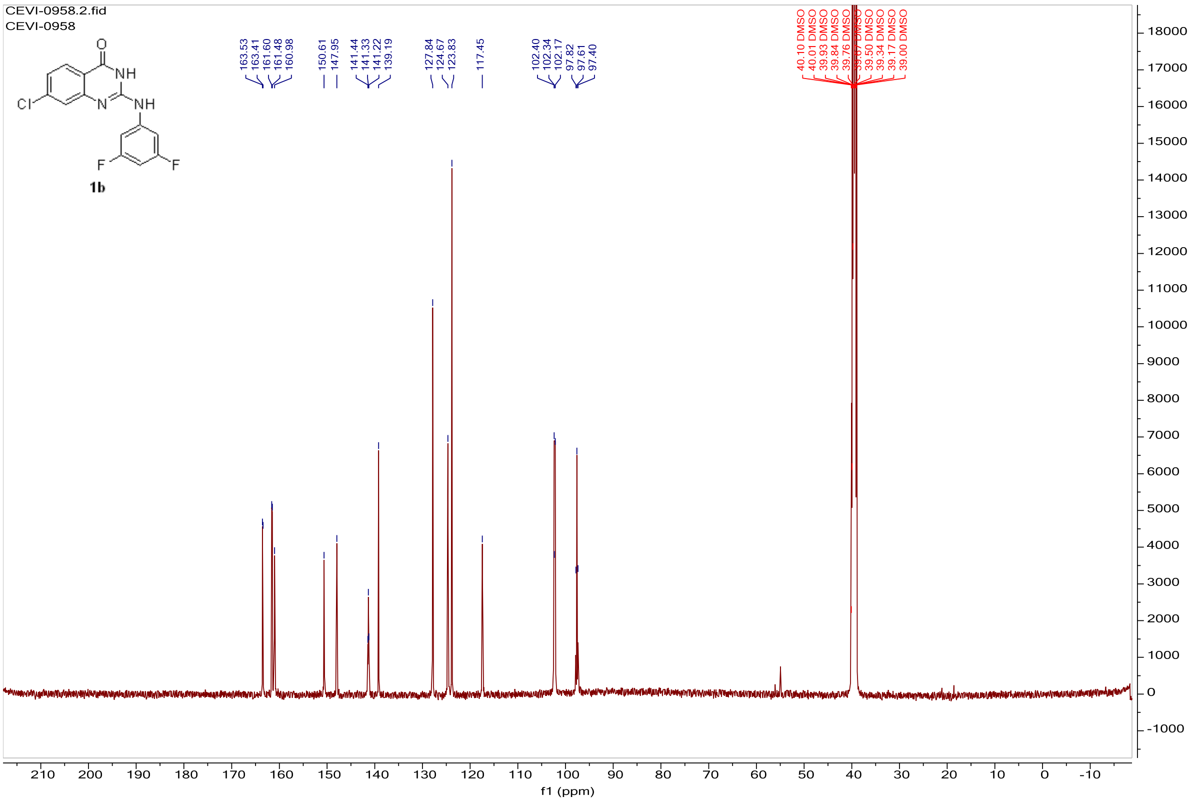

^{13}C NMR spectrum (125 MHz, DMSO-d_6) of **1b**

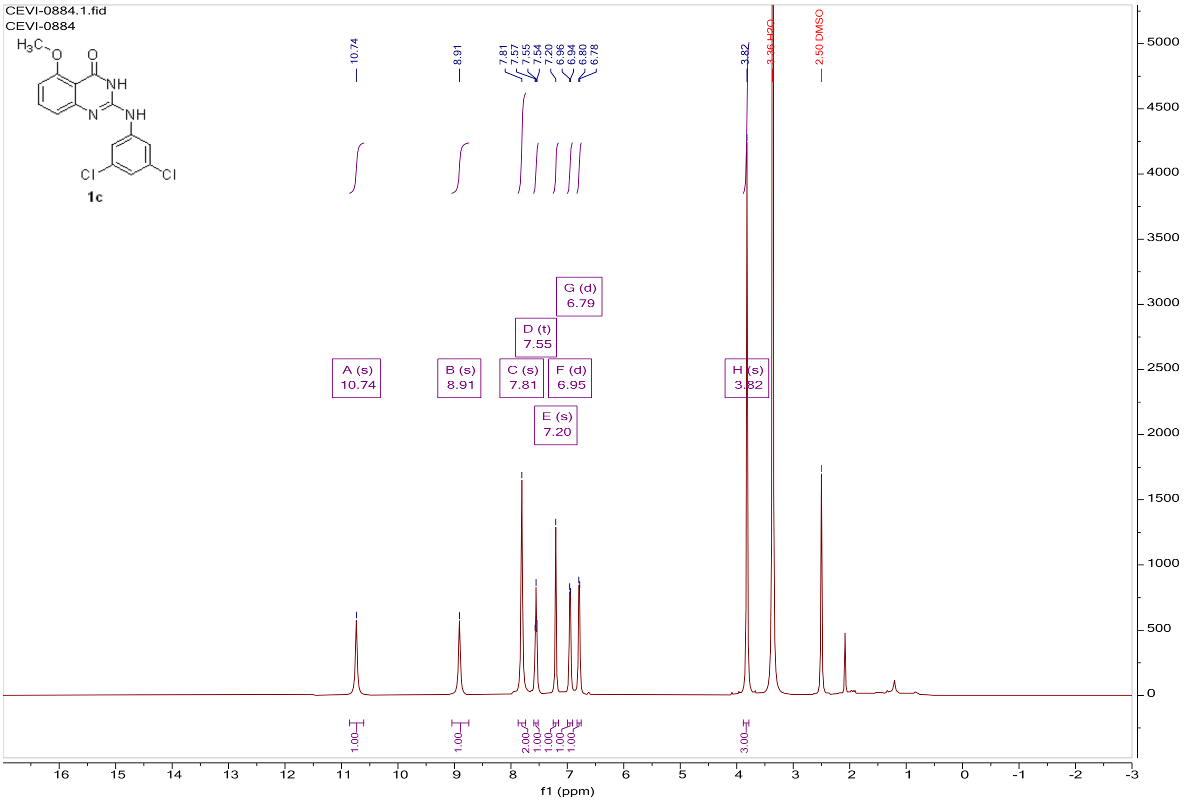

^{1}H NMR spectrum (500 MHz, DMSO-d_6) of **1c**

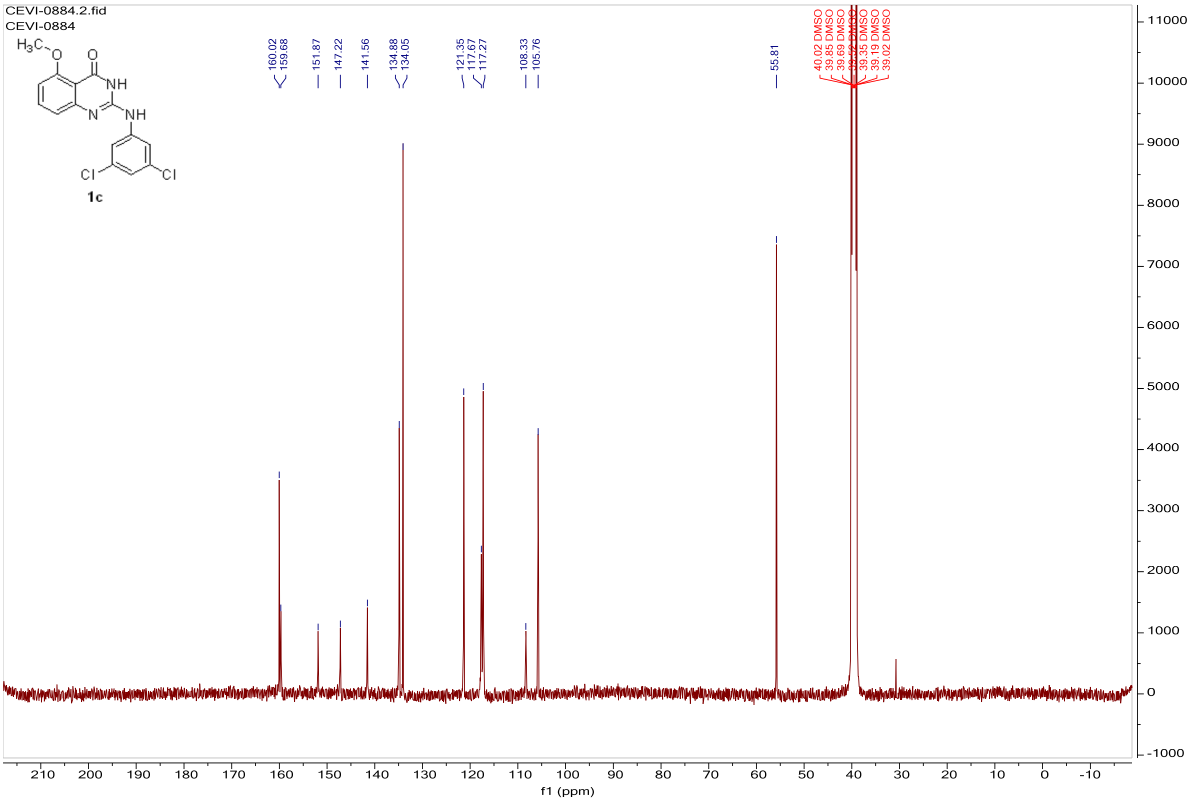

^{13}C NMR spectrum (125 MHz, DMSO-d_6) of **1c**

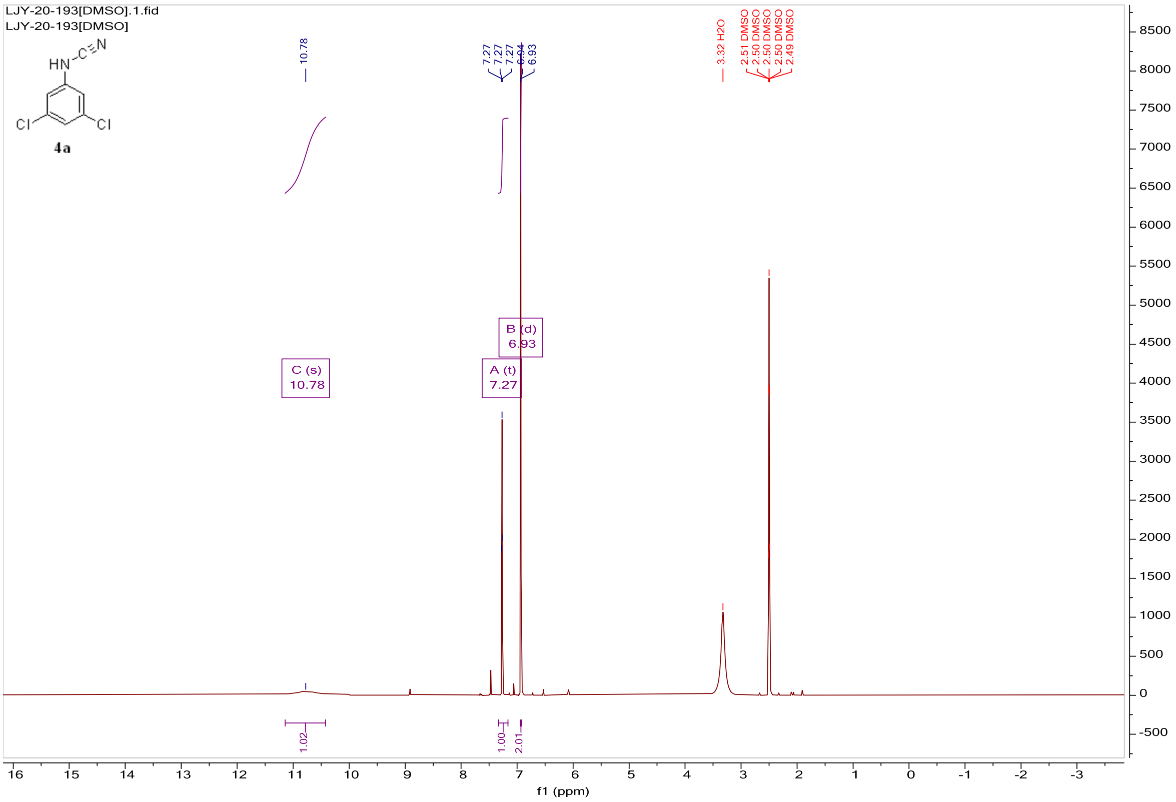

^{1}H NMR spectrum (400 MHz, DMSO-d_6) of **4a**

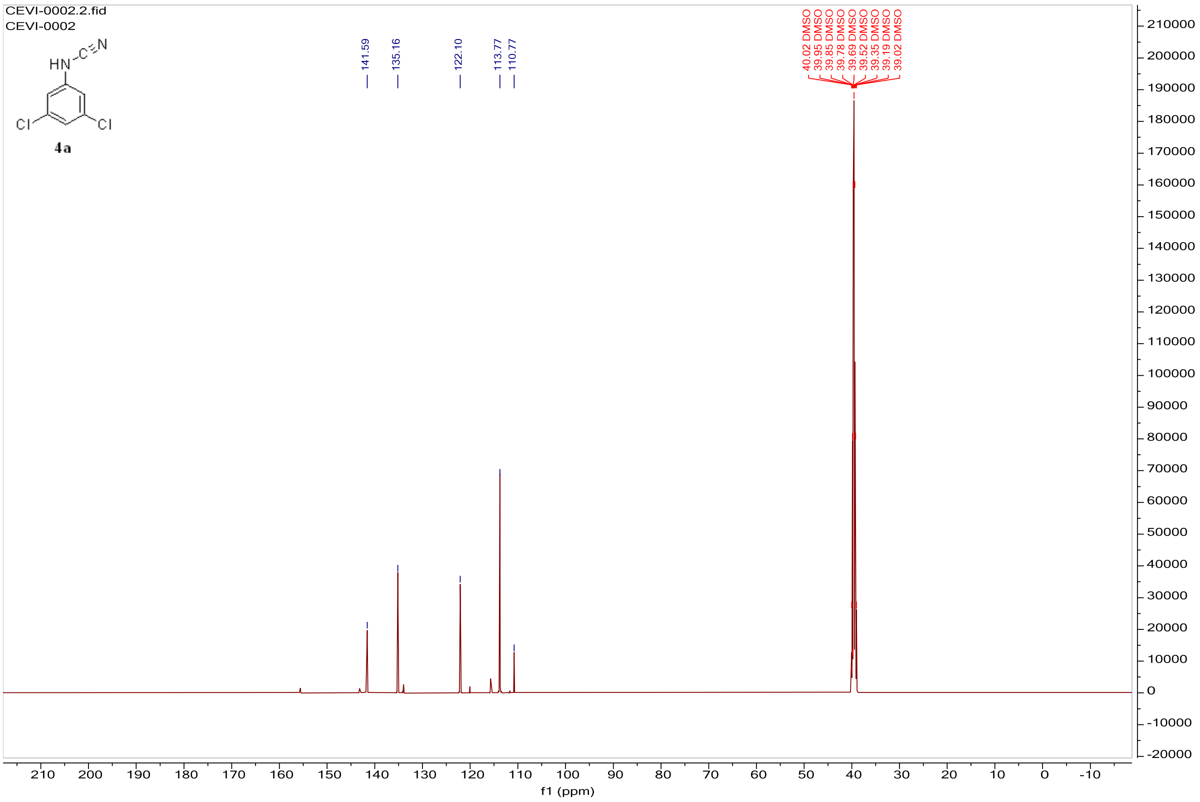

^{13}C NMR spectrum (100 MHz, DMSO-d_6) of **4a**

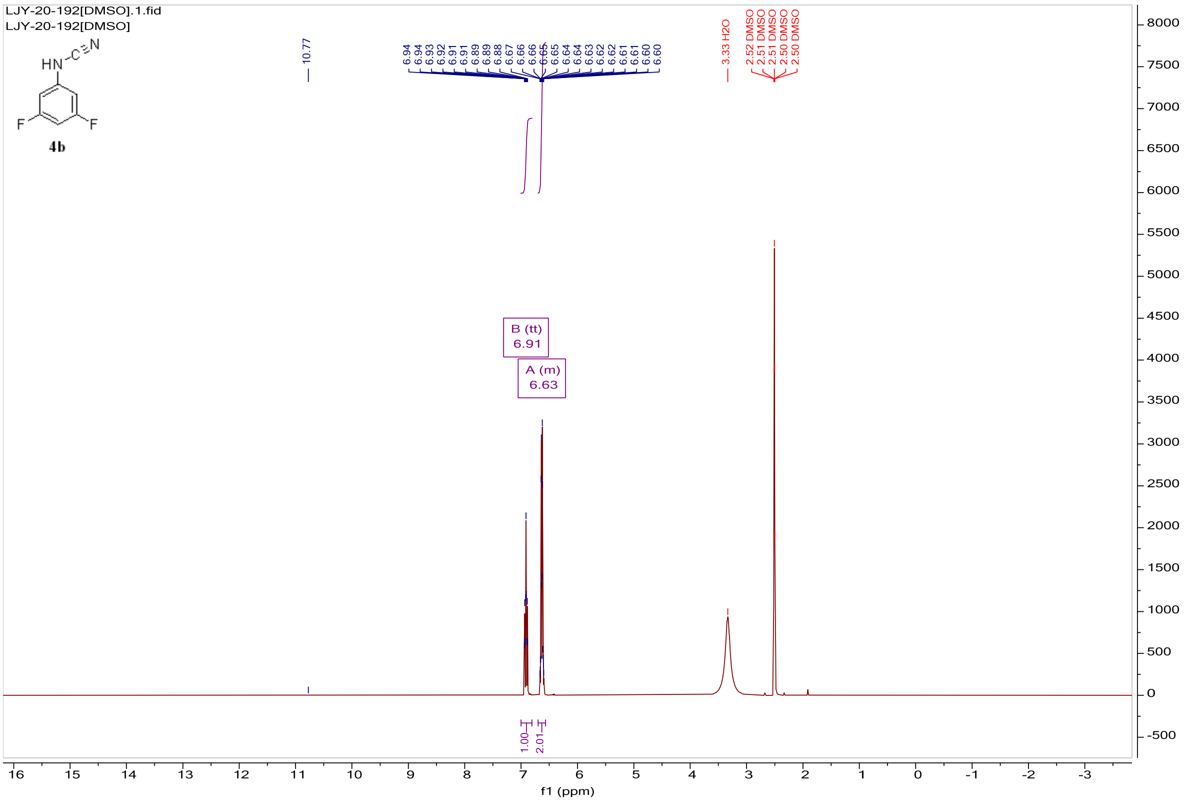

^{1}H NMR spectrum (400 MHz, DMSO-d_6) of **4b**

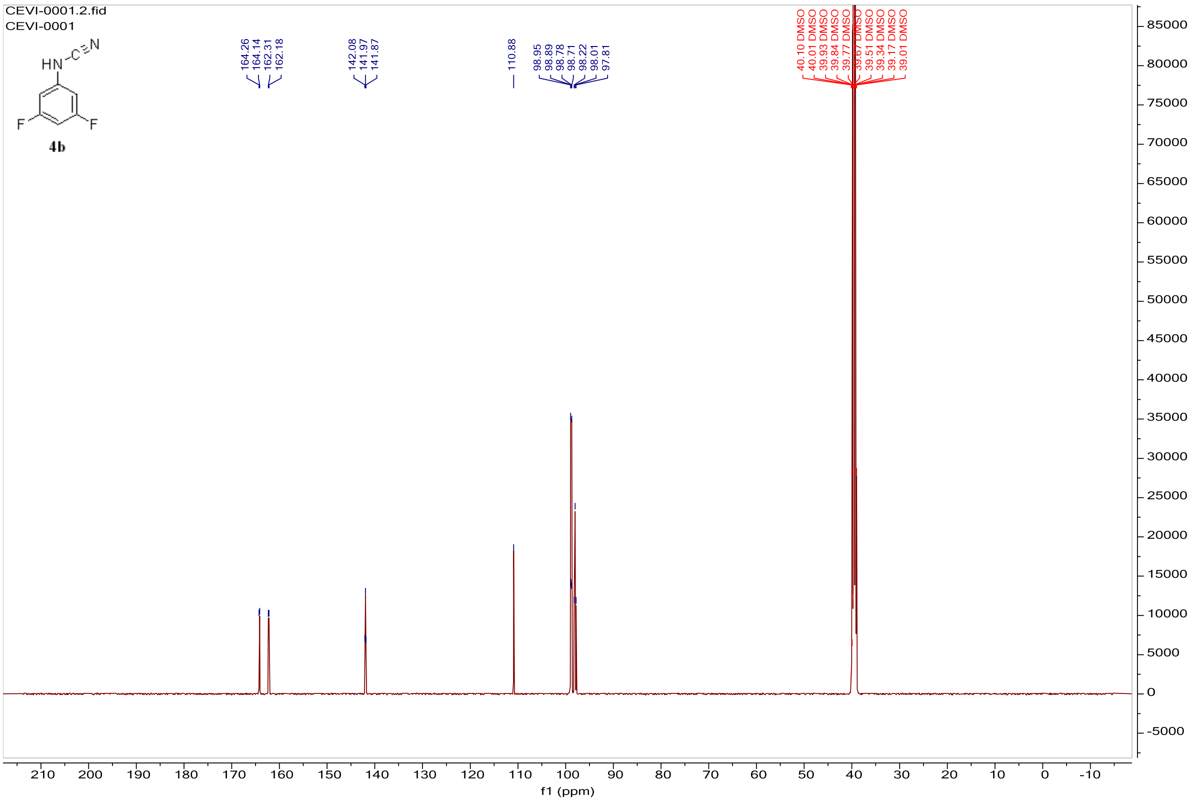

^{13}C NMR spectrum (100 MHz, DMSO-d_6) of **4b**

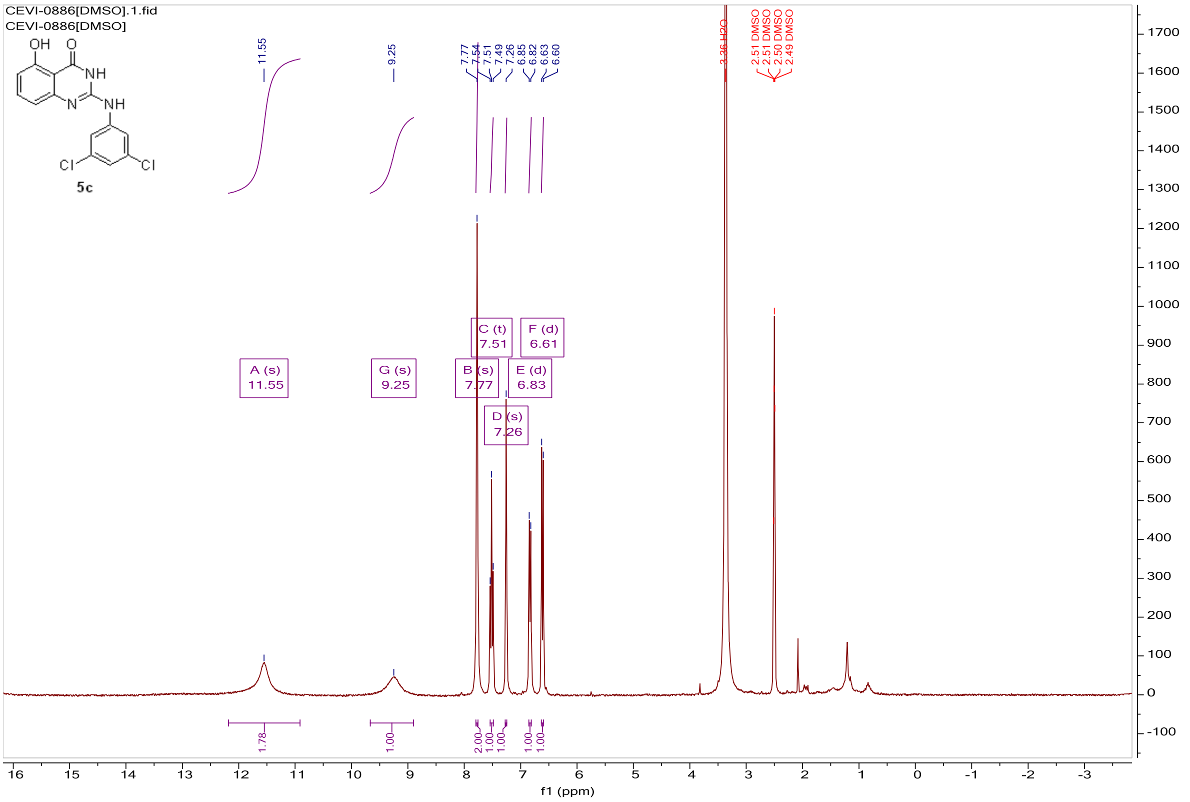

^{1}H NMR spectrum (300 MHz, DMSO-*d*6) of **5c**

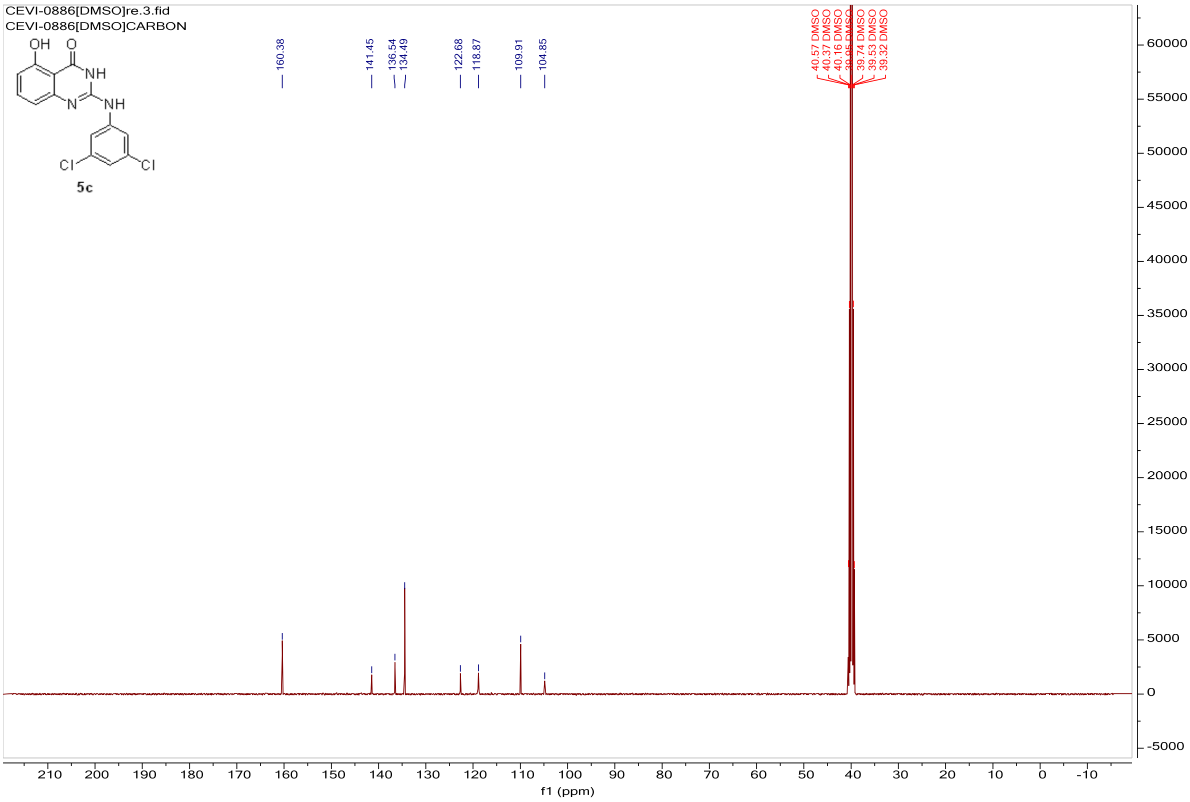

^{13}C NMR spectrum (125 MHz, DMSO-*d*6) of **5c**

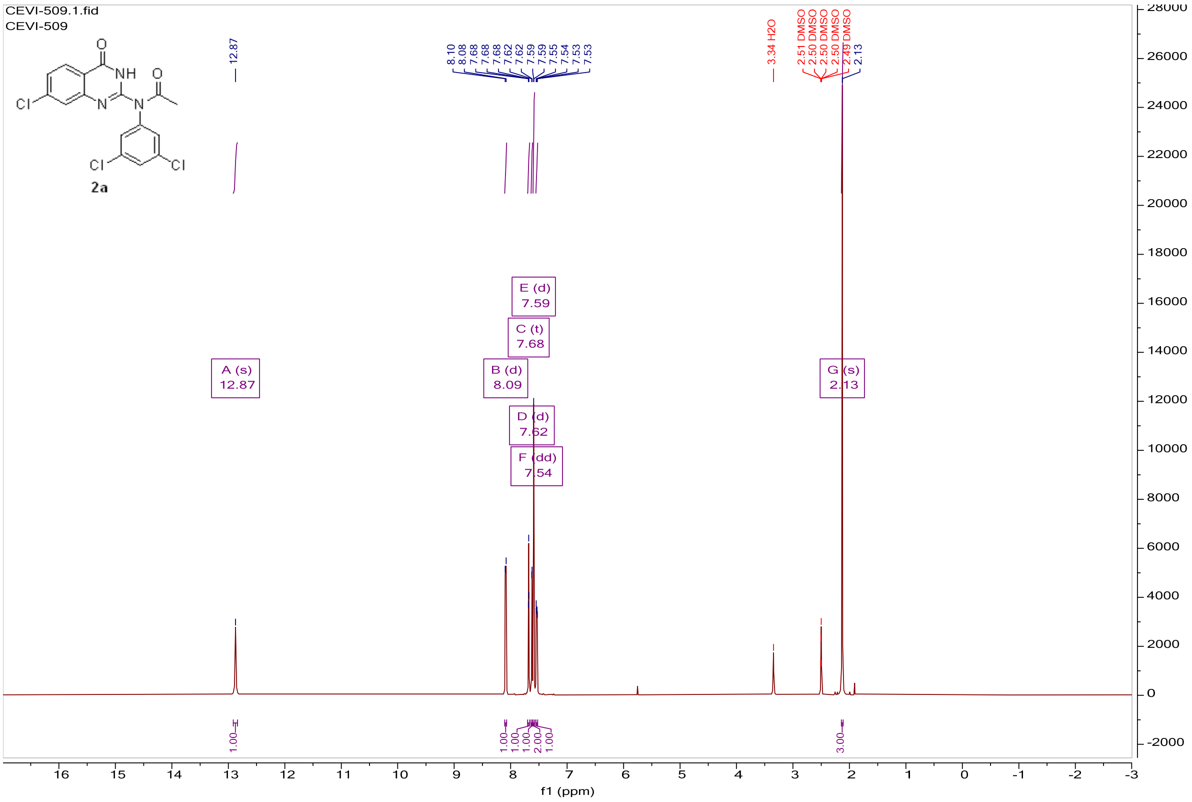

^{1}H NMR spectrum (500 MHz, DMSO-d_6) of **2a**

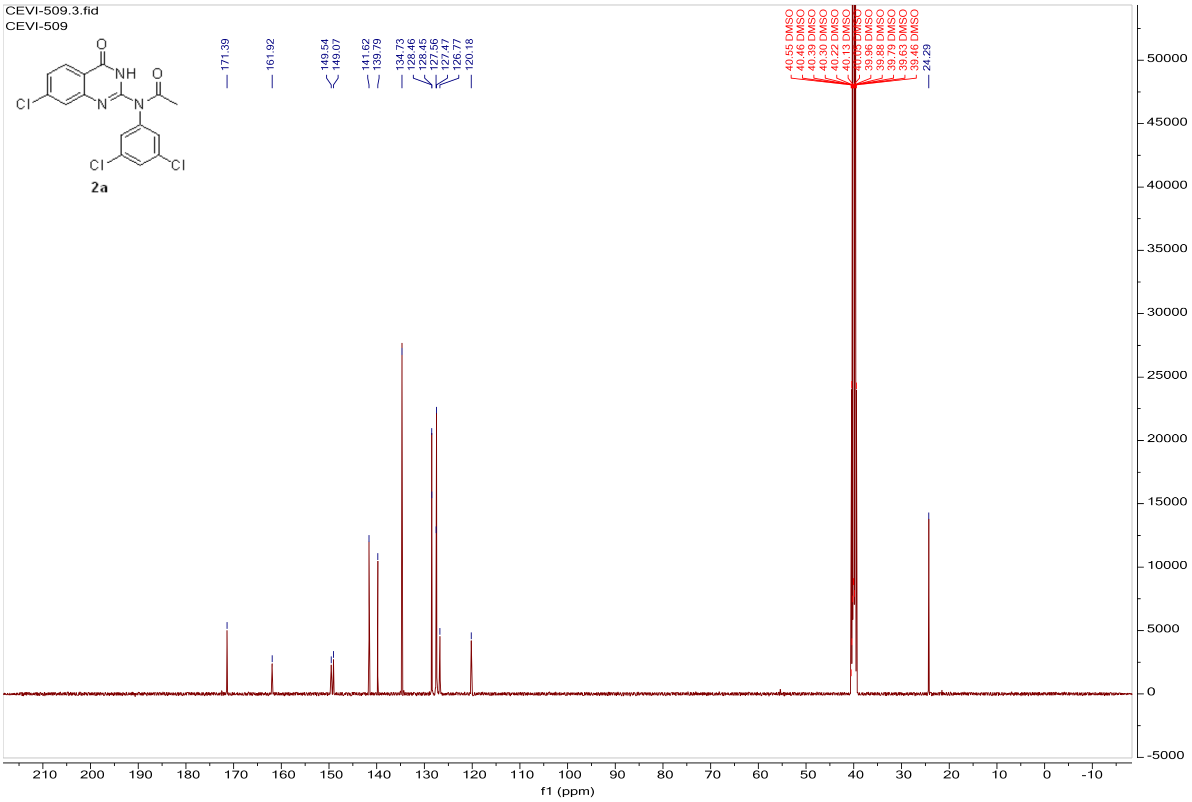

^{13}C NMR spectrum (125 MHz, DMSO-d_6) of **2a**

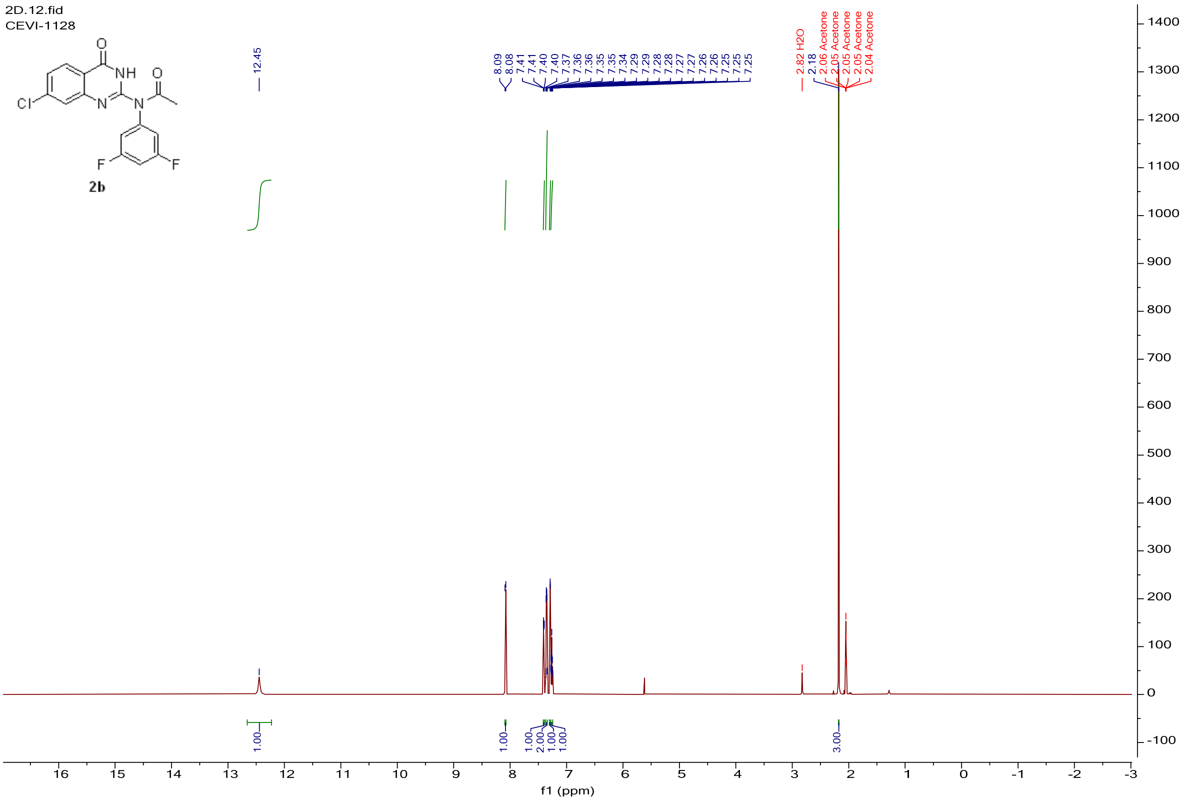

^{1}H NMR spectrum (700 MHz, Acetone-d_6) of **2b**

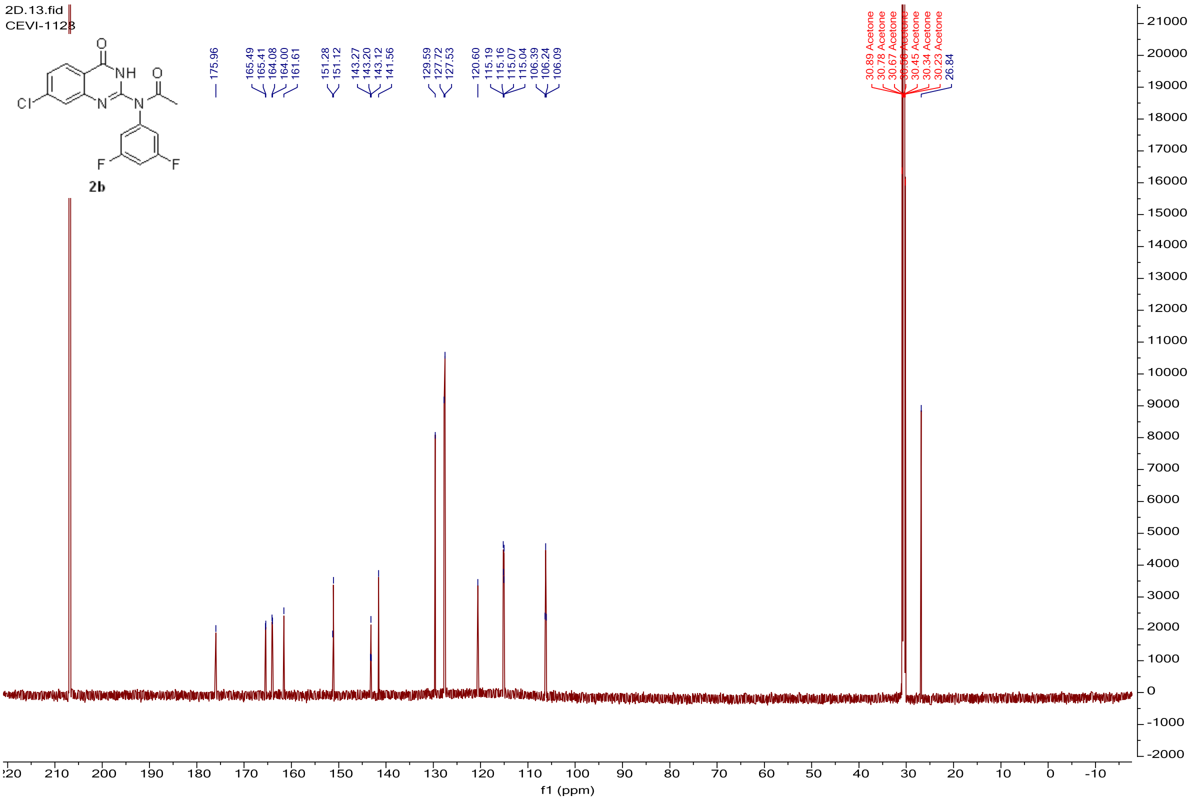

^{13}C NMR spectrum (175 MHz, Acetone-d_6) of **2b**

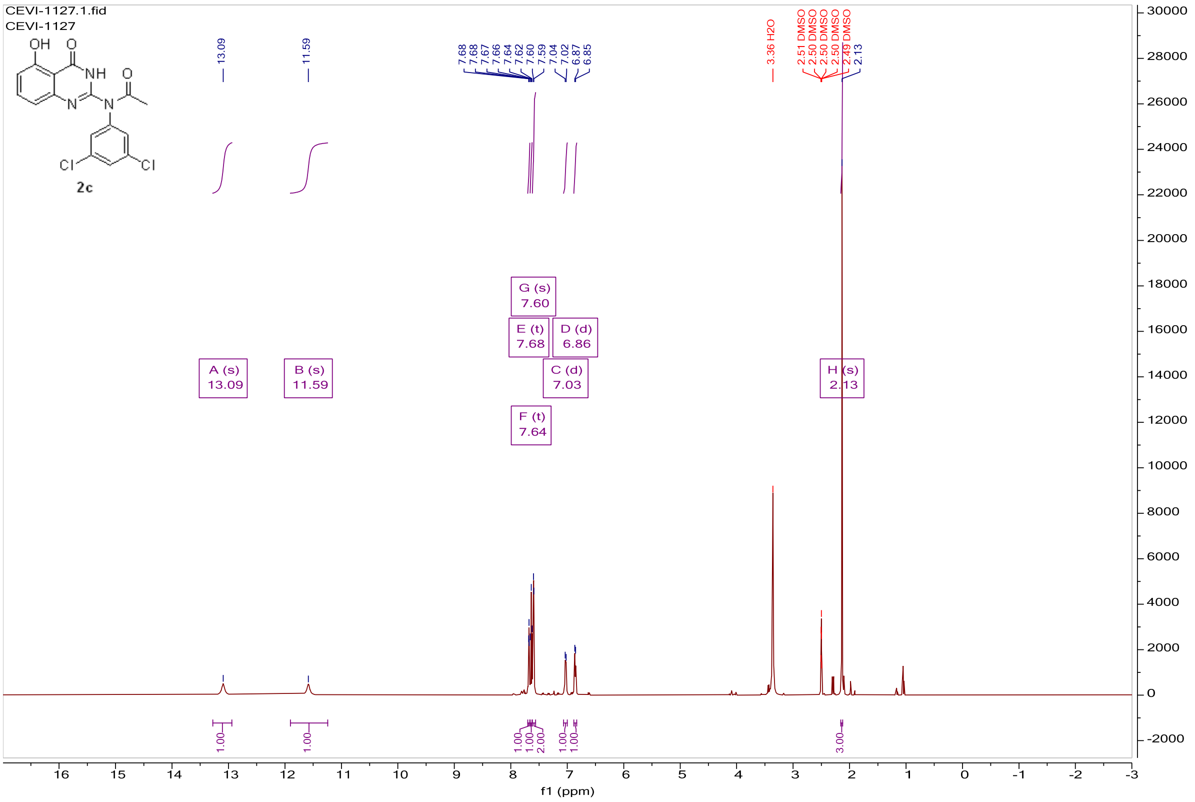

^{1}H NMR spectrum (500 MHz, DMSO-d_6) of **2c**

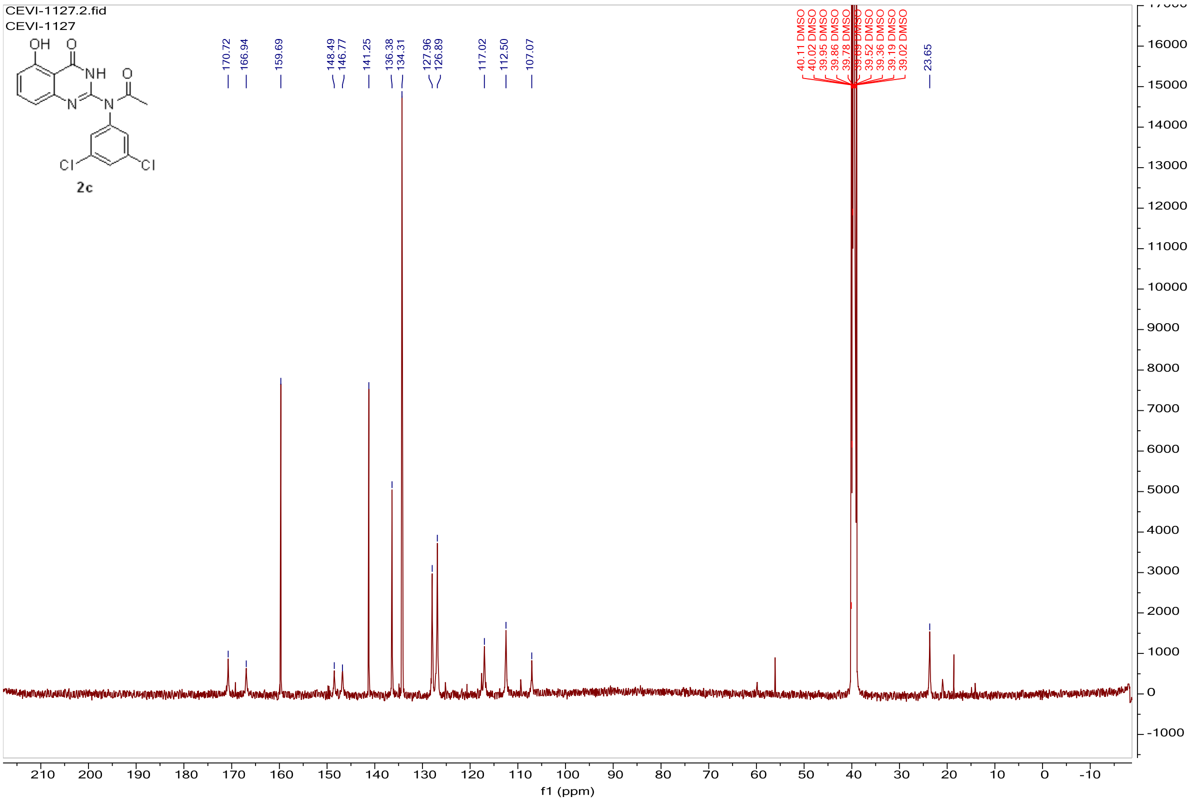

^{13}C NMR spectrum (125 MHz, DMSO-d_6) of **2c**

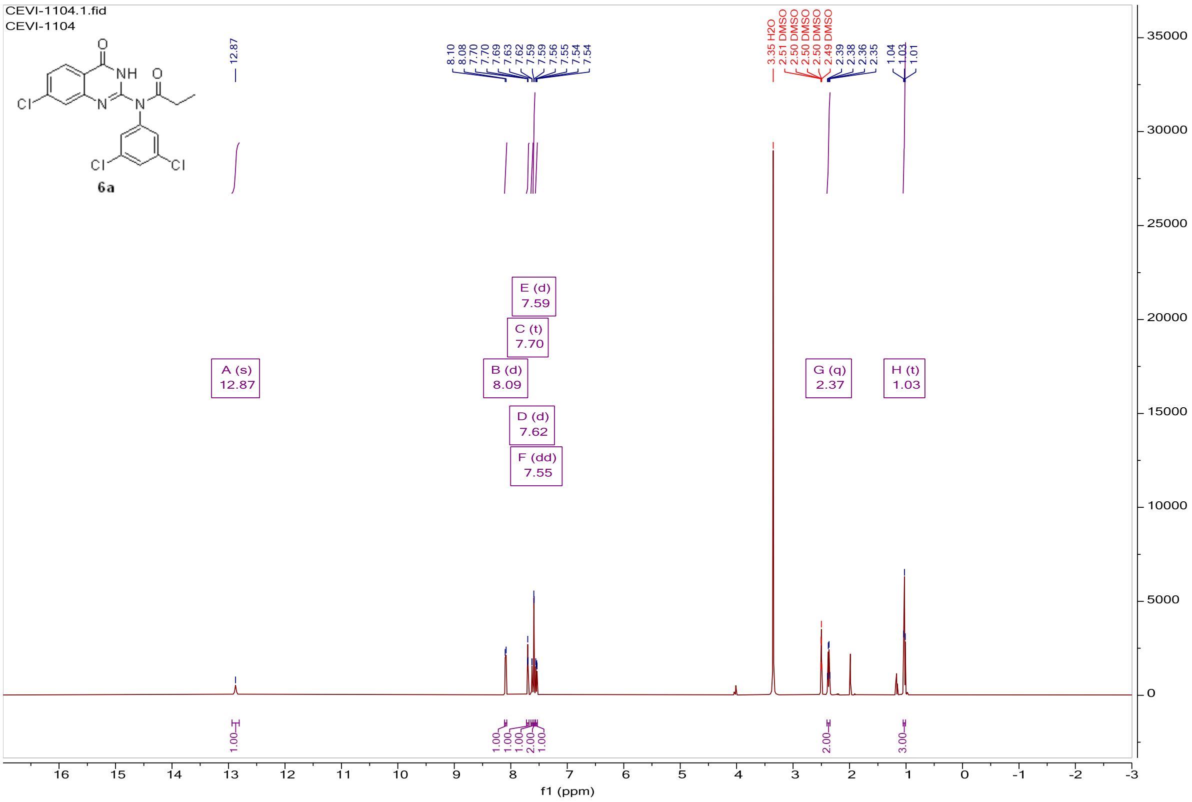

^{1}H NMR spectrum (500 MHz, DMSO-*d*$_6$) of **6a**

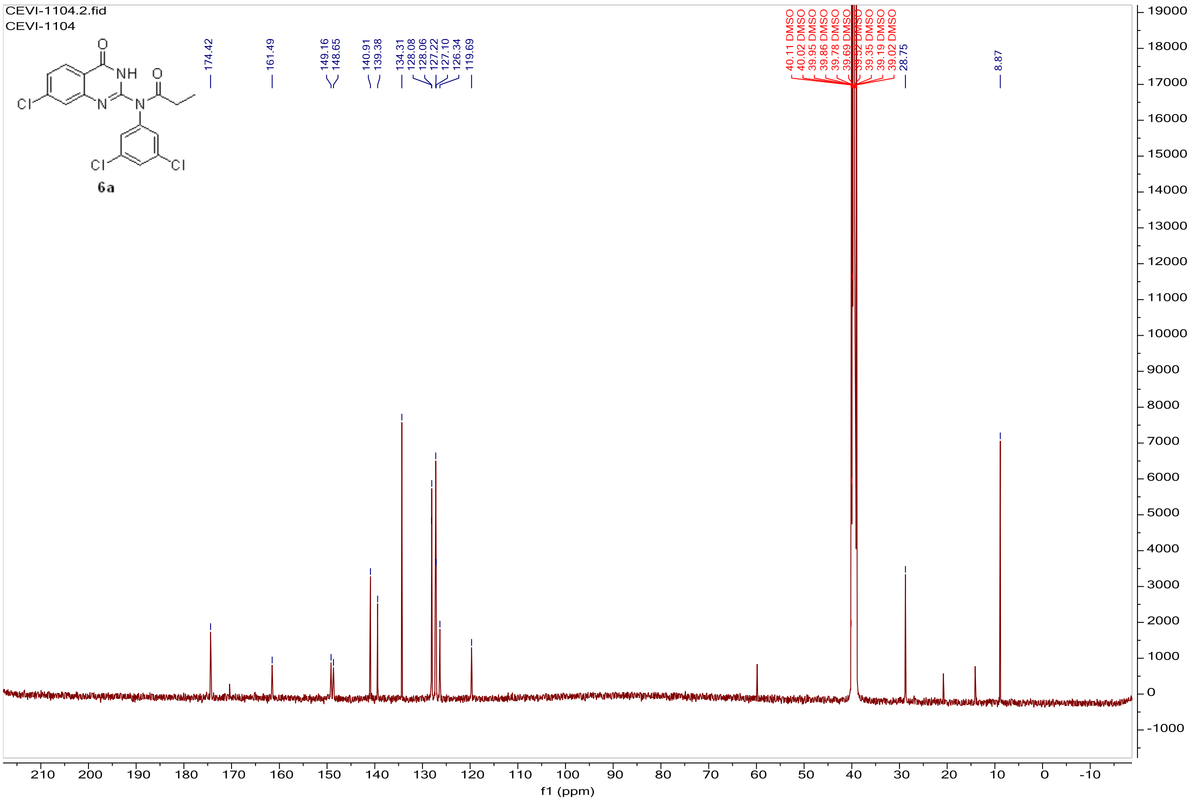

^{13}C NMR spectrum (125 MHz, DMSO-*d*$_6$) of **6a**

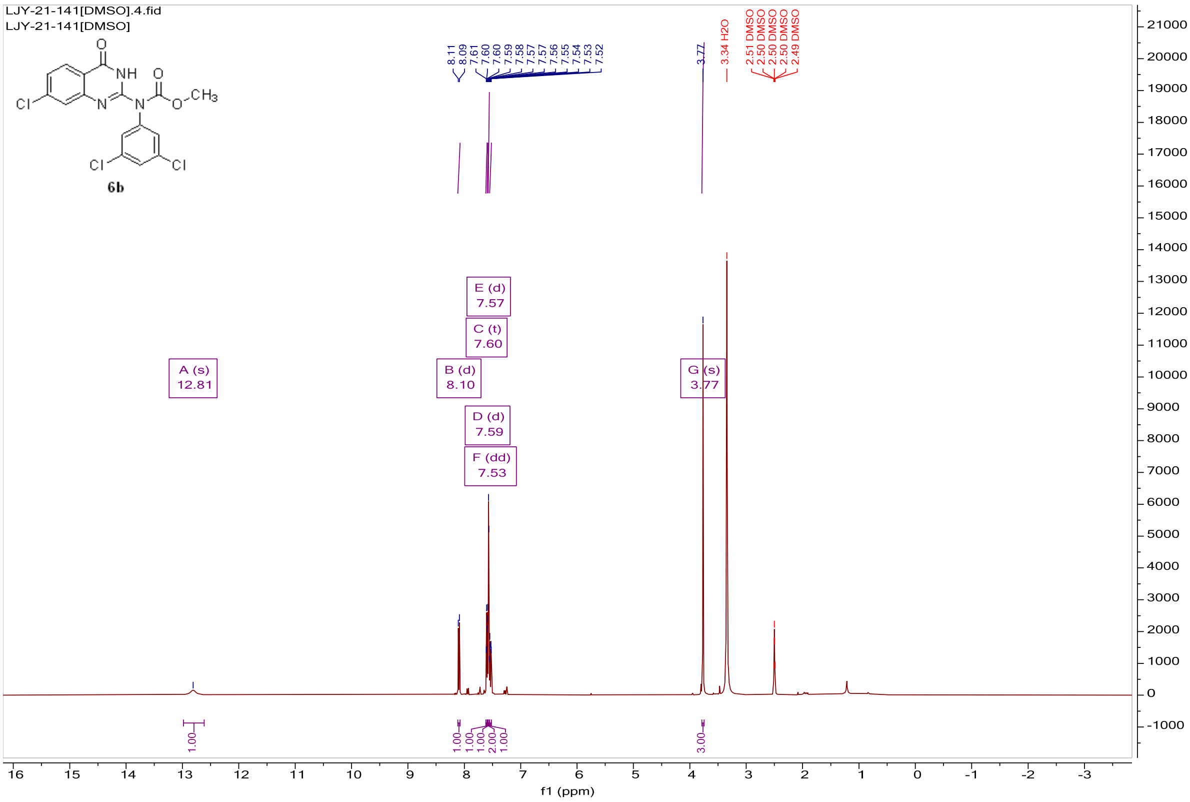

^{1}H NMR spectrum (400 MHz, DMSO-d_6) of **6b**

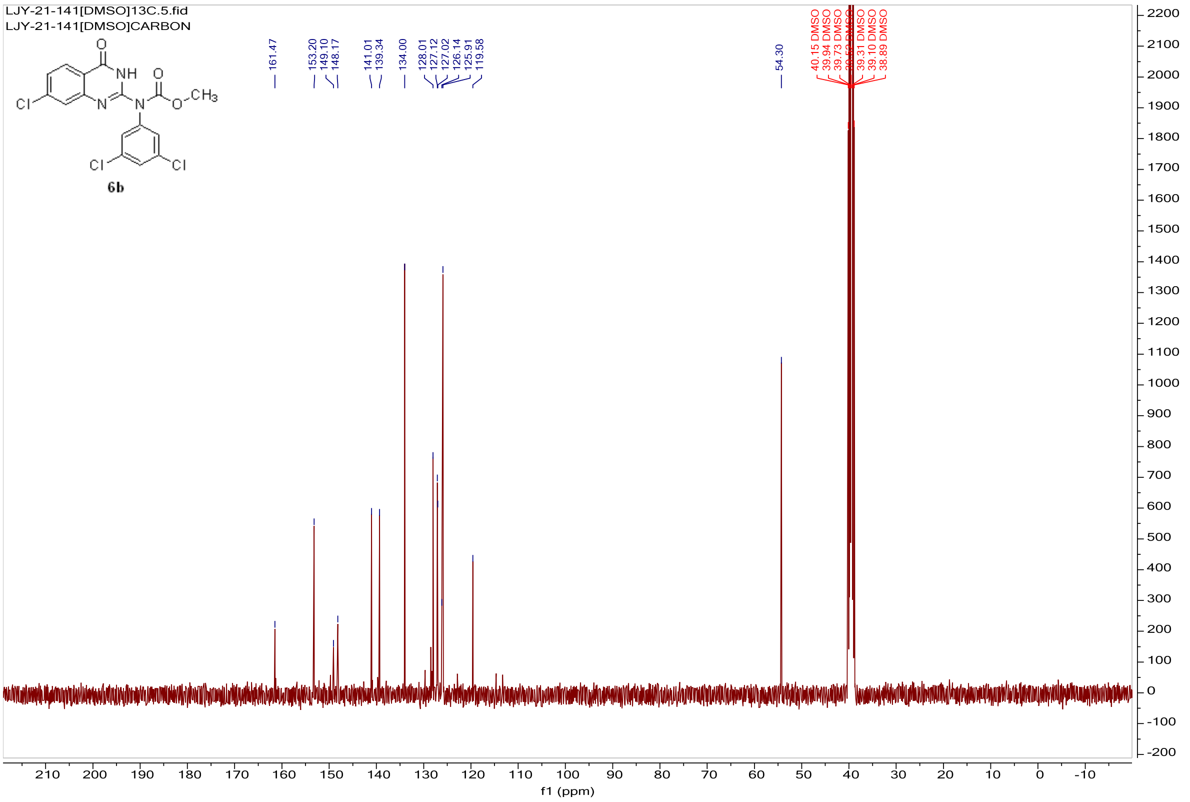

^{13}C NMR spectrum (100 MHz, DMSO-d_6) of **6b**

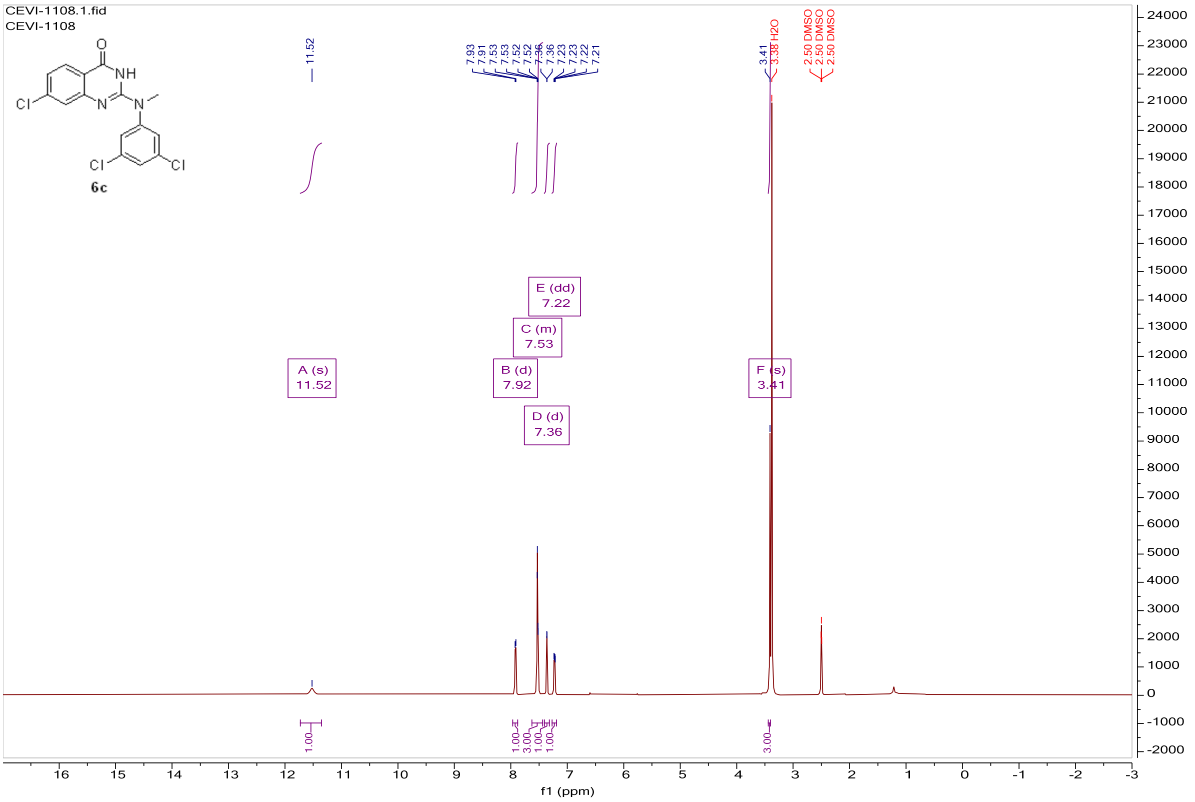

^{1}H NMR spectrum (500 MHz, DMSO-d_6) of **6c**

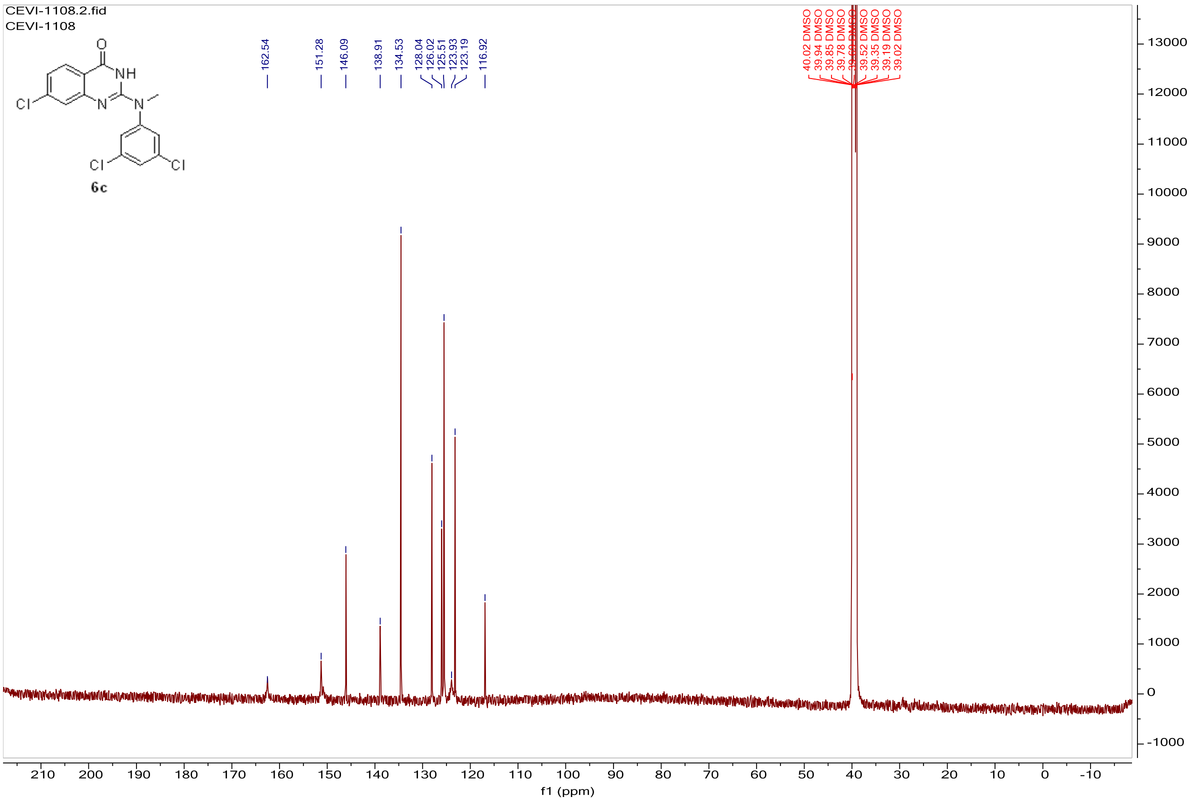

^{13}C NMR spectrum (125 MHz, DMSO-d_6) of **6c**

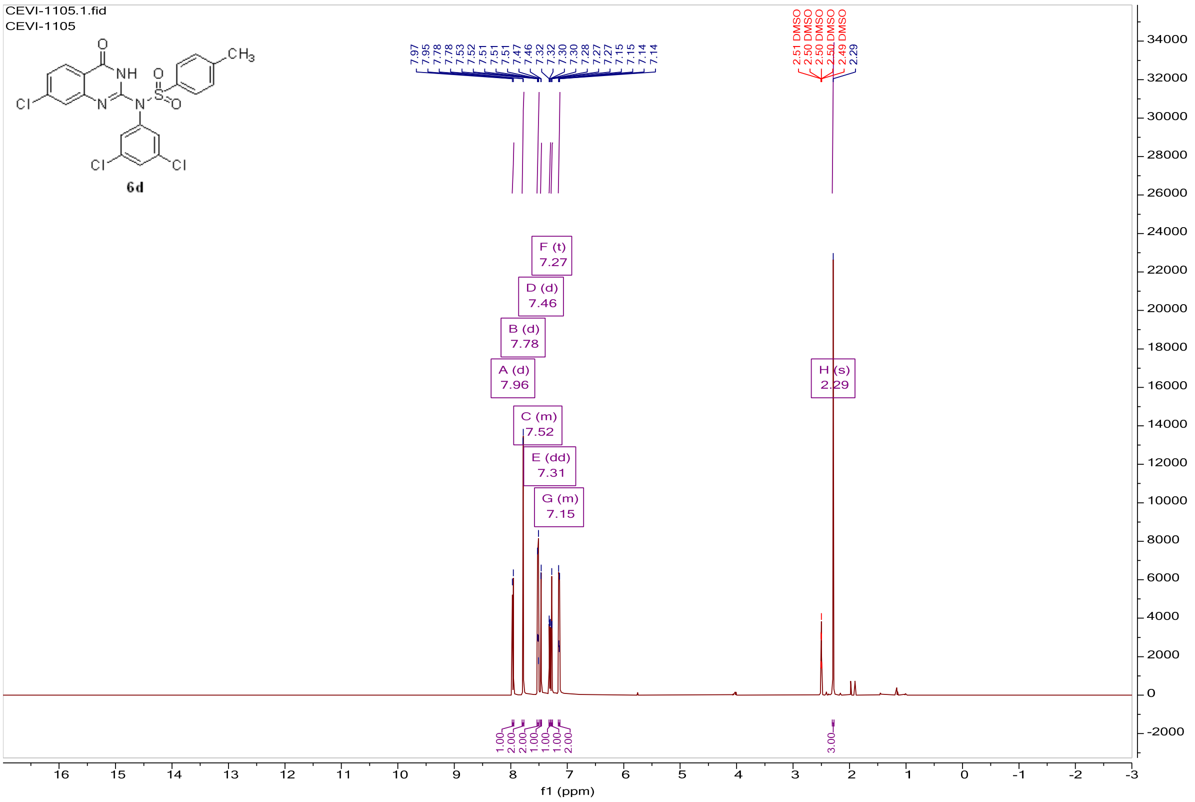

^{1}H NMR spectrum (500 MHz, DMSO-d_6) of **6d**

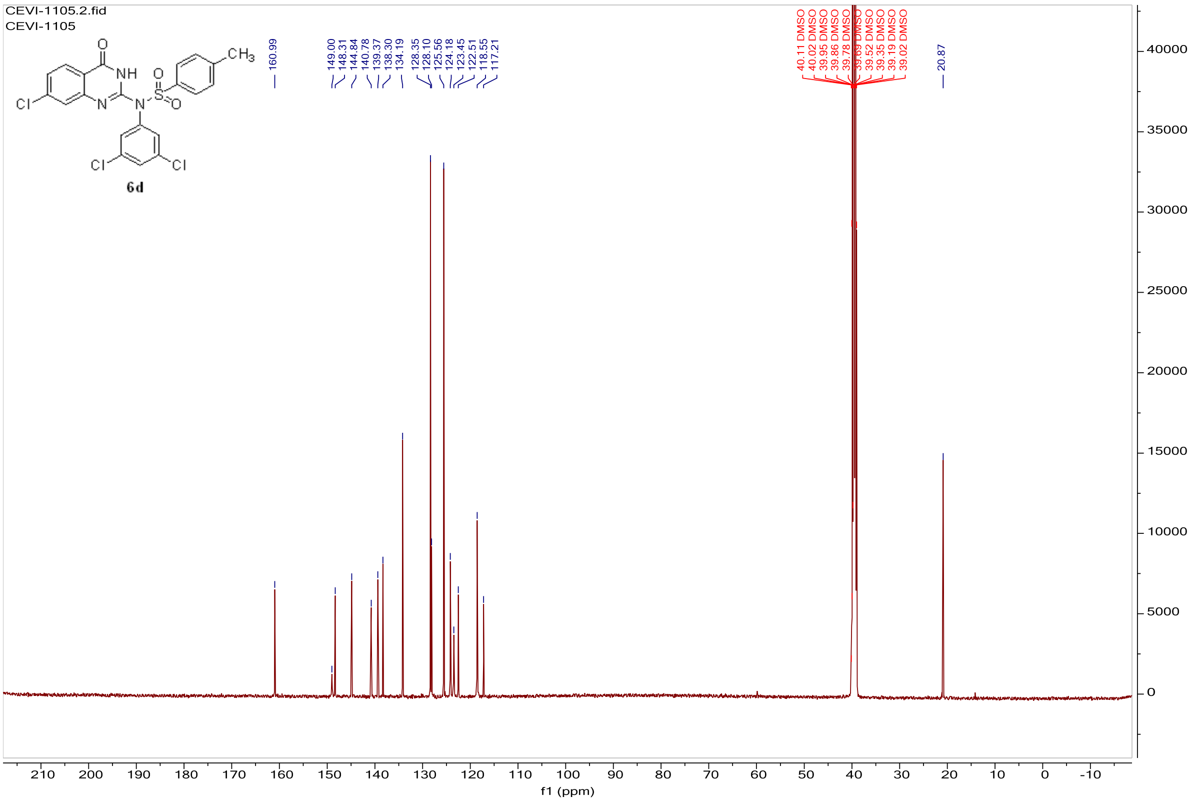

^{13}C NMR spectrum (125 MHz, DMSO-d_6) of **6d**

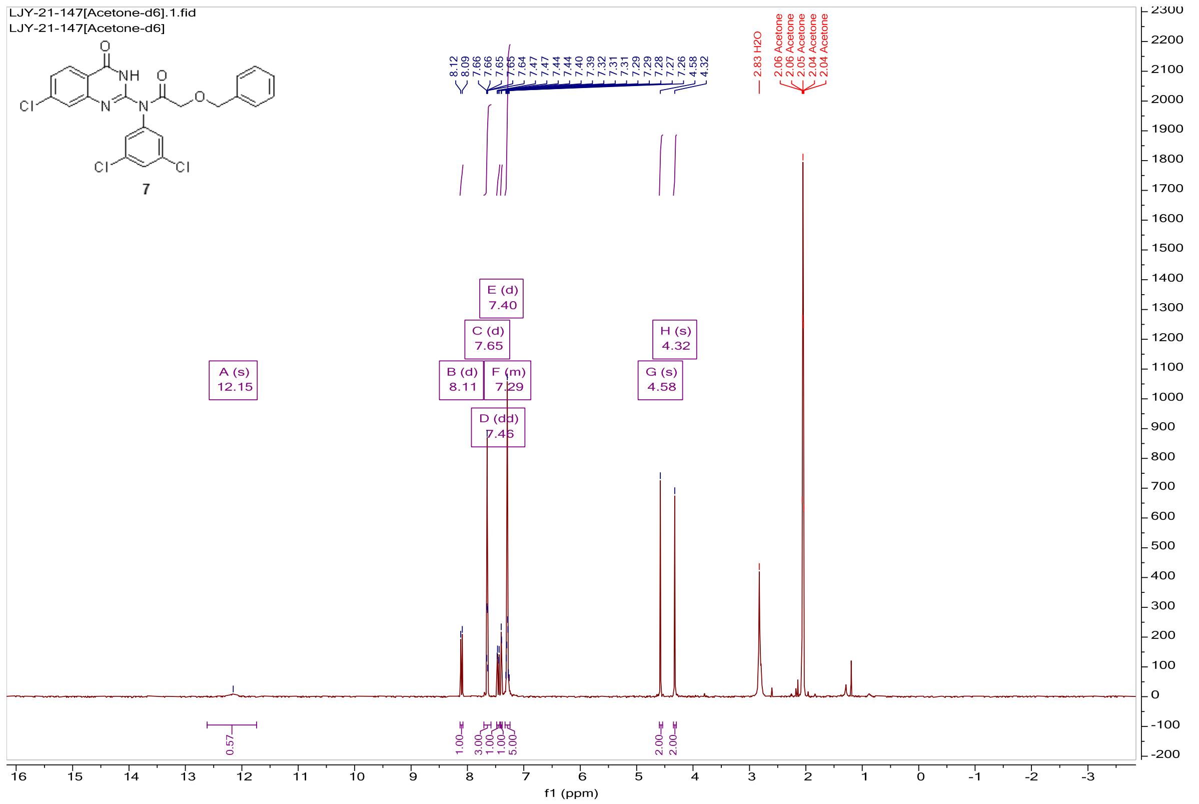

^{1}H NMR spectrum (300 MHz, Acetone-d_6) of **7**

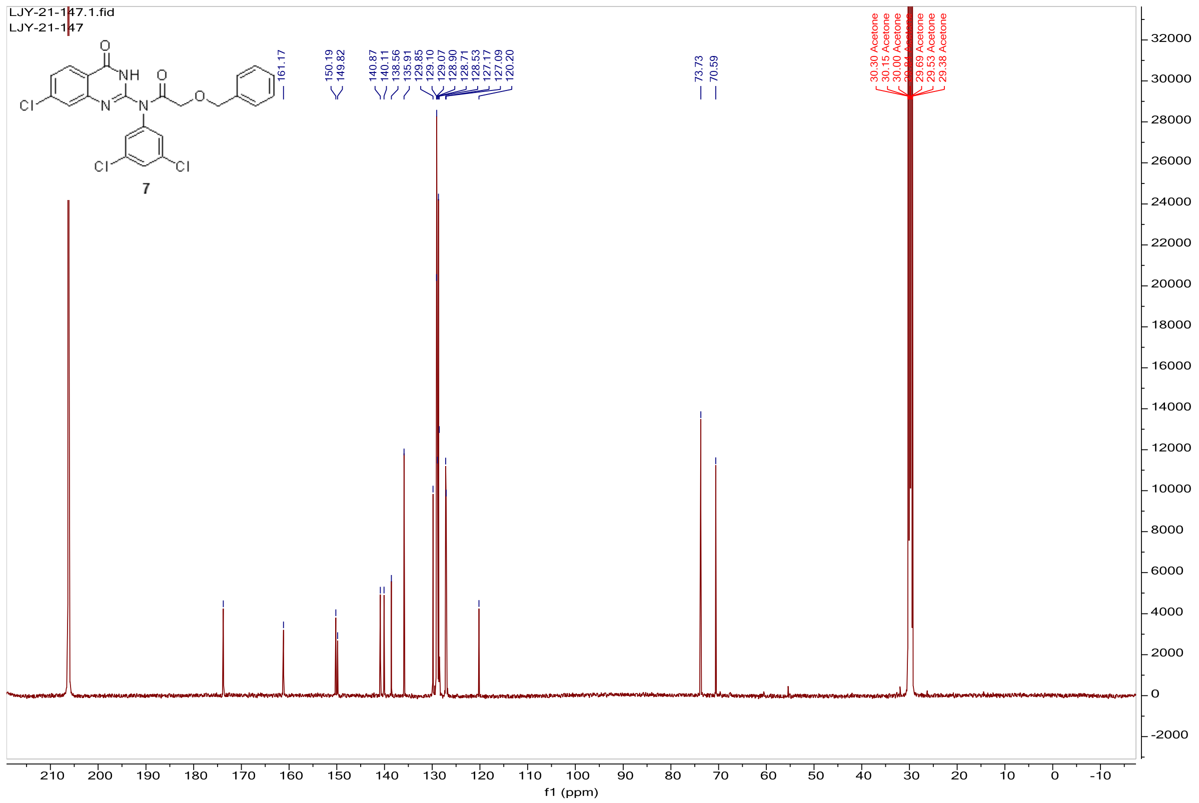

^{13}C NMR spectrum (125 MHz, Acetone-d_6) of **7**

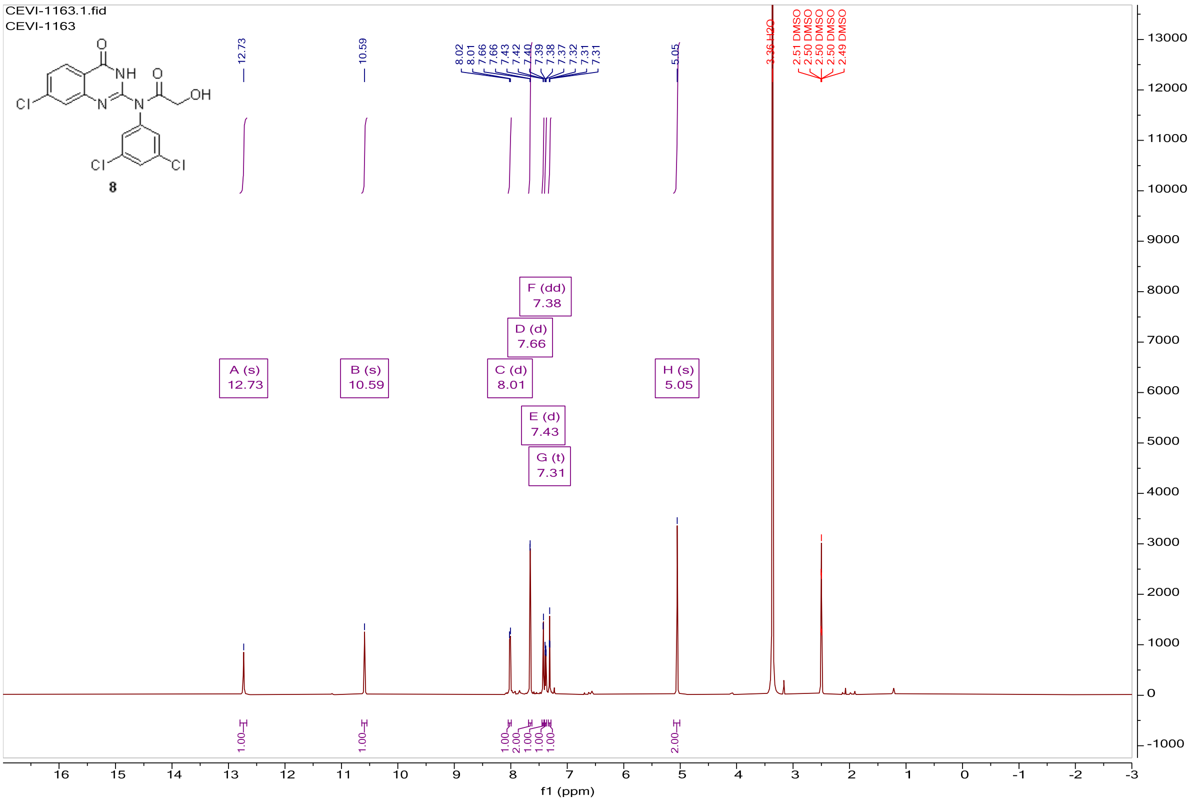

^{1}H NMR spectrum (500 MHz, DMSO-d_6) of **8**

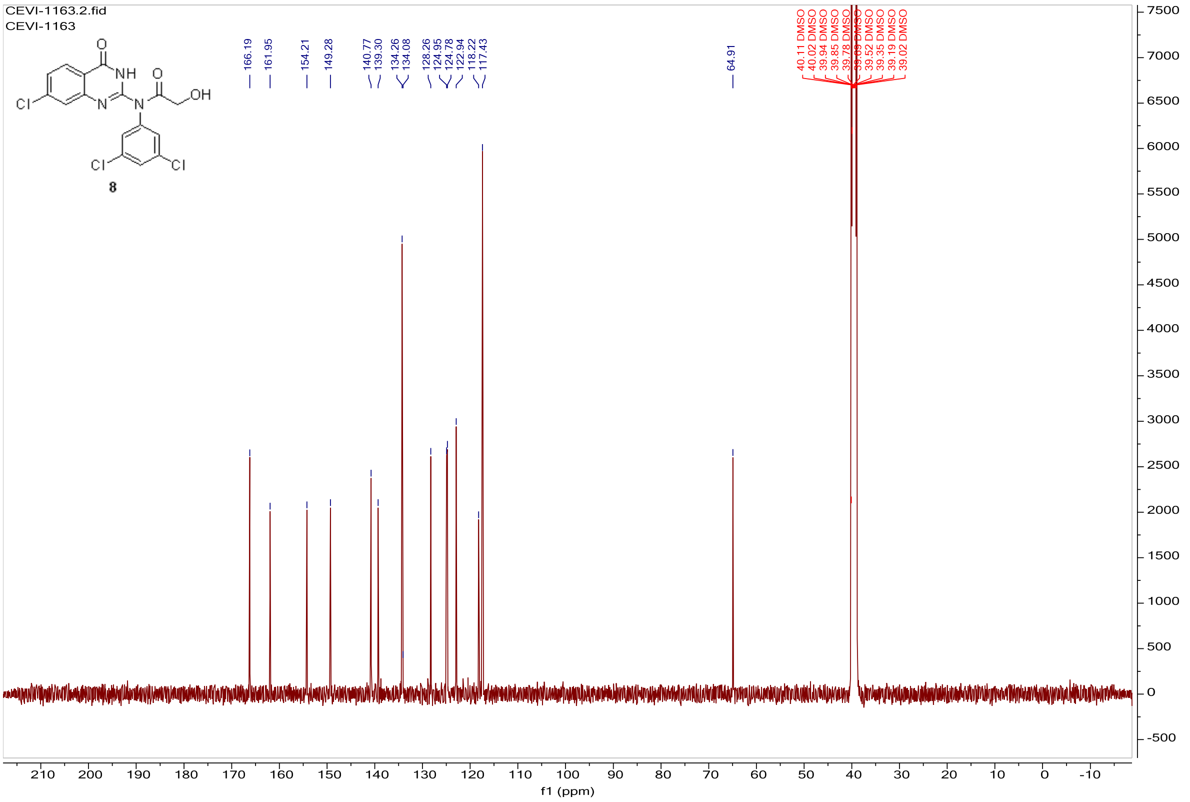

^{13}C NMR spectrum (125 MHz, DMSO-d_6) of **8**

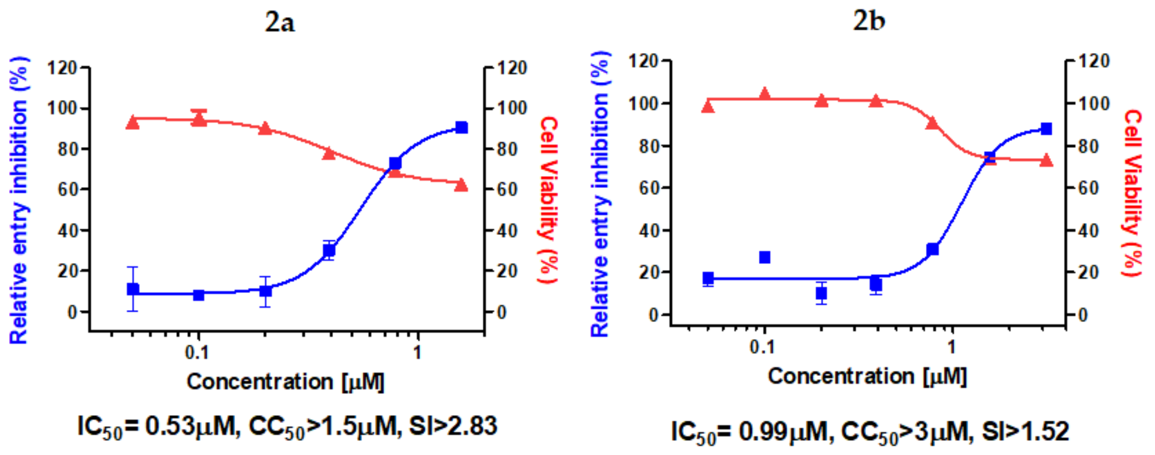

Figure 1S. Concentration-response inhibition curves of **2a** and **2b** against SARS-CoV-2 pseudovirus
